# SARS-CoV-2 nsp13, nsp14, nsp15 and orf6 function as potent interferon antagonists

**DOI:** 10.1080/22221751.2020.1780953

**Published:** 2020-06-20

**Authors:** Chun-Kit Yuen, Joy-Yan Lam, Wan-Man Wong, Long-Fung Mak, Xiaohui Wang, Hin Chu, Jian-Piao Cai, Dong-Yan Jin, Kelvin Kai-Wang To, Jasper Fuk-Woo Chan, Kwok-Yung Yuen, Kin-Hang Kok

**Affiliations:** a State Key Laboratory of Emerging Infectious Diseases, The University of Hong Kong, Pokfulam, Hong Kong Special Administrative Region, People’s Republic of China; b Department of Microbiology, Li Ka Shing Faculty of Medicine, The University of Hong Kong, Pokfulam, Hong Kong Special Administrative Region, People’s Republic of China; c Carol Yu Centre for Infection, The University of Hong Kong, Pokfulam, Hong Kong Special Administrative Region, People’s Republic of China; d School of Biomedical Sciences, Li Ka Shing Faculty of Medicine, The University of Hong Kong, Pokfulam, Hong Kong Special Administrative Region, People’s Republic of China; e Department of Clinical Microbiology and Infection Control, The University of Hong Kong-Shenzhen Hospital, Shenzhen, People’s Republic of China; f Department of Microbiology, Queen Mary Hospital, Pokfulam, Hong Kong Special Administrative Region, People’s Republic of China

**Keywords:** COVID-19, SARS-CoV-2, interferon antagonist, PLpro, orf6

## Abstract

The Coronavirus disease 2019 (COVID-19), which is caused by the novel SARS-CoV-2 virus, is now causing a tremendous global health concern. Since its first appearance in December 2019, the outbreak has already caused over 5.8 million infections worldwide (till 29 May 2020), with more than 0.35 million deaths. Early virus-mediated immune suppression is believed to be one of the unique characteristics of SARS-CoV-2 infection and contributes at least partially to the viral pathogenesis. In this study, we identified the key viral interferon antagonists of SARS-CoV-2 and compared them with two well-characterized SARS-CoV interferon antagonists, PLpro and orf6. Here we demonstrated that the SARS-CoV-2 nsp13, nsp14, nsp15 and orf6, but not the unique orf8, could potently suppress primary interferon production and interferon signalling. Although SARS-CoV PLpro has been well-characterized for its potent interferon-antagonizing, deubiquitinase and protease activities, SARS-CoV-2 PLpro, despite sharing high amino acid sequence similarity with SARS-CoV, loses both interferon-antagonising and deubiquitinase activities. Among the 27 viral proteins, SARS-CoV-2 orf6 demonstrated the strongest suppression on both primary interferon production and interferon signalling. Orf6-deleted SARS-CoV-2 may be considered for the development of intranasal live-but-attenuated vaccine against COVID-19.

## Introduction

The newly emerged severe acute respiratory syndrome coronavirus 2 (SARS-CoV-2) that causes the COVID-19 pandemic is a novel coronavirus phylogenetically close to the SARS-CoV and bat-related SARS-CoV [1,2]. Although SARS-CoV and SARS-CoV-2 both belong to the same genus betacoronavirus and cause severe upper respiratory illness in humans, their clinical presentation and immune responses are different from each other [3]. One of the unique features of this COVID-19 epidemic is the presence of many asymptomatic patients and the observation of significant viral shedding during incubation period [4,5]. Furthermore, serial viral load profile showed that the viral load of COVID-19 peaks around the time of symptom onset or at clinical presentation which is different from that of SARS or MERS when their viral load peak at about 7–10 days after symptom onset [6,7]. These factors may contribute to the high efficiency of viral transmission. It is believed that immunosuppression at the cellular level is one of the strategies applied by SARS-CoV-2 during the early phase of infection. Recently, ex-vivo SARS-CoV-2 infection study using human lung tissue explant culture provided clues about such immunosuppression [8]. When compared to SARS-CoV, SARS-CoV-2 replicated much more efficiently in both type I and II pneumocytes and alveolar macrophages in ex-vivo lung tissues. However, SARS-CoV-2 induced less pro-inflammatory cytokines and chemokines. More potent suppression of both type-I/III and type-II interferons has also been observed in this ex-vivo tissue culture model. SARS-CoV has been shown to effectively suppress interferon production during early infection, and the delayed interferon signalling contributes to the exuberant inflammatory response and severe lung immunopathology [9]. Further investigation on the innate immune suppression by SARS-CoV-2 in-vivo will elucidate the viral pathogenesis of COVID-19 disease.

Innate interferon signalling is the first line of defence against various viral infections. Viral pathogen-associated molecular patterns (PAMPs), such as viral genomes or viral mRNAs will be first recognized by host pattern recognition receptors (PRR) including Toll-like receptors and RIG-I like receptors, followed by the activation of downstream signalling molecules such as adaptor proteins MAVS and MyD88, kinases TBK1 and IKK epsilon, and transcription factors IRF3 and NFĸB, resulting in the strong production of type-I/III interferons. This is referred as primary interferon production. Secreted interferons function as autocrine or paracrine, turning on the antiviral status in infected or neighbouring cells respectively. Subsequent activation of kinases Tyk2 and Jak1, and the downstream transcriptional complex comprising STAT1/2 and IRF9, activates the interferon-stimulated response element (ISRE)-containing promoter, hence induces the transcription of the antiviral genes or interferon-stimulated genes (ISGs) [10,11]. ISGs such as OAS and IFITM have been shown to inhibit coronavirus infection through degradation of viral RNAs and restriction of viral entry [12,13]. Altogether, both the Type-I/III interferon production and the subsequent signalling are critical for the control of early coronavirus infection.

In order to establish successful infection, many viruses have evolved with mechanisms to suppress host innate immune activation. Coronaviruses are known to express multiple interferon antagonists for optimal inhibition of interferon signalling. For instance, nsp1, papain-like protease (PLpro), nsp7, nsp15, orf3b, M, orf6 and N proteins of SARS-CoV have all been shown to possess interferon-antagonizing properties [14–27]. Nsp14 and nsp16, which are N-7- and 2-Oʹ-methyltransferase respectively, cap the viral RNAs so that they can evade immune recognition by PRR and IFIT1 [28–31]. In addition, two independent studies clearly demonstrated that SARS-CoV PLpro and orf6 are the key interferon antagonists during infection. Infection of recombinant SARS-CoV with either the substitution of bat PLpro or deletion of orf6 resulted in compromised interferon antagonism or induction of interferon production [24,32]. Given the pivotal role of interferon signalling on coronavirus infection, there is an urgent need to better understand the interferon antagonism by this novel and galloping pandemic SARS-CoV-2.

In this study, we identified SARS-CoV-2 nsp13 (helicase), nsp14 (exonuclease), nsp15 (endoribonuclease), and accessory protein orf6 as the most potent viral interferon antagonists. The activity of PLpro and orf6 derived from SARS-CoV and SARS-CoV-2 have been further compared. While orf6 remained equally effective in inhibiting interferon signalling, we found that SARS-CoV-2 PLpro loses the interferon-antagonizing and deubiquitinase activity. With the natural loss of interferon-antagonizing property of PLpro, further deletion of orf6 shall render the SARS-CoV-2 virus highly interferon-sensitive. Such recombinant virus may serve as a plausible vaccine candidate.

## Materials and methods

### Cell culture, transfection and Sendai virus infection

293FT cells (Thermo Fisher Scientific) was cultured in Dulbecco's Modified Eagle Medium (DMEM) supplemented with 10% Foetal bovine serum (FBS). Cell were seeded 12–18 h before transfection. DNA and GeneJuice (Novagen) were diluted with Opti-MEM (Thermo Fisher Scientific) and mixed at 1:3 ratio before transfection. Sendai virus (Cantell strain) was purchased from American Type Culture Collection (ATCC) and further propagated in 10-day-old Specific-pathogen-free (SPF) eggs (SPAFAS). Cells were infected with indicated haemagglutinating units of Sendai virus in DMEM supplemented with 10% FBS.

### Plasmids

Gene fragments of all 27 viral proteins (except PLpro of SARS-CoV-2) were gene synthesized (Sangon Biotech) with codon optimization. PLpro and orf6 of both SARS-CoV and SARS-CoV-2 were PCR amplified from cDNA reverse-transcribed from viral RNA extracted from viral supernatant. All gene fragments were cloned into pCAGEN expression vector with c-terminal FLAG-tag and confirmed by Sanger sequencing. Plasmids used have previously been detailed [33]. pEBG plasmid containing the GST-tagged RIG-I N-terminal card domains was used. Interferon-beta firefly luciferase reporter (IFNβ-luc), which comprises the promoter of IFNβ, is a kind gift of Professor Takashi Fujita, Kyoto University. Interferon-stimulated response element firefly luciferase reporter (ISRE-luc) which contains five repeats of ISRE enhancer element upstream of minimal TA promoter was purchased from Clontech. Renilla luciferase reporter (pRL-TK) that contains an HSV-thymidine kinase promoter was purchased from Promega.

### Dual Luciferase assay

293FT cells seeded in 24-well plates were transfected in triplicate with either IFNβ-luc or ISRE-luc firefly luciferase reporter and pRL-TK Renilla luciferase reporter (Promega), together with the indicated combination of expression plasmids. At indicated timepoint, cells were lysed using 1X Passive lysis buffer (Promega). Firefly and Renilla luciferase signals were quantified using Dual Luciferase Reporter Assay System (Promega), represented as relative luciferase activity by dividing firefly luciferase signal with Renilla luciferase signal and presented in log10 scale. Statistical significance between two selected groups was calculated by unpaired two-tailed student’s t-test. Error bars represent standard deviation of three biological replicates.

### Western blotting, immunostaining and antibodies

For western blotting, cells were lysed with RIPA lysis buffer (150 mM NaCl, 50 mM Tris-HCl pH8.0, 0.1% SDS, 1% NP-40, 0.5% sodium deoxycholate) containing EDTA-free protease inhibitor cocktail (Roche). After centrifugation, clear supernatant was collected, added with protein sample buffer to final concentration of 50 mM Tris-HCl pH6.8, 2% SDS, 10% glycerol, 30 mM DTT, 0.002% bromophenol blue, boiled for 10 min and separated on SDS-PAGE gel. Proteins were semi-dry transferred onto PVDF membrane, blocked with 5% skim milk and probed with antibodies as indicated. For immunostaining, cells seeded on chamber-slides were fixed with 4% paraformaldehyde, 0.5% NP-40 permeabilized and blocked with 5% normal donkey serum (Jackson ImmunoResearch), followed by staining using the indicated antibodies. Images were captured using LSM880 confocal microscope (ZEISS). Mouse anti-FLAG-tag (Sigma-Aldrich), anti-beta-actin (Sigma-Aldrich), anti-GAPDH (Santa Cruz Biotechnology) and rabbit anti-HA-tag (Cell Signaling Technology), anti-IRF3 (Santa Cruz Biotechnology) antibodies were used.

### RNA extraction, reverse transcription and quantitative PCR

Cells were lysed with RNAiso Plus reagent (Takara) and total RNA was extracted following manufacturer’s protocol. RNA was reverse transcribed using PrimeScript RT reagent Kit with gDNA Eraser (Takara) using the provided random hexamer plus oligo-dT primer mix. Transcript abundance were quantitated by quantitative PCR using SYBR Premix Ex Taq (Takara). The expression level of an indicated gene was represented as relative expression in comparison to that of GAPDH house-keeping gene. Statistical significance between two selected groups was calculated by unpaired two-tailed student’s *t*-test. Error bars represent standard deviation of replicates of three.

### Bead-based cytokine assay

Cell culture supernatant was collected at indicated timepoint and stored at −80°C until quantitation. Secreted IFN-α2, IFN-β, IFN-λ1 and IFN-λ2/3 in 25 µl supernatant were quantitated using LEGENDplex Human Anti-Virus Response Panel (13-plex) kit (BioLegend) following manufacturers’ instruction. Samples were analysed by flow cytometry using BD LSR Fortessa Cell Analyzer (BD Biosciences). Statistical significance between two selected groups was calculated by unpaired two-tailed student’s t-test. Error bars represent standard deviation of replicates of three.

## Results

### Screening of putative interferon antagonists encoded by SARS-CoV-2

**Figure 1.**
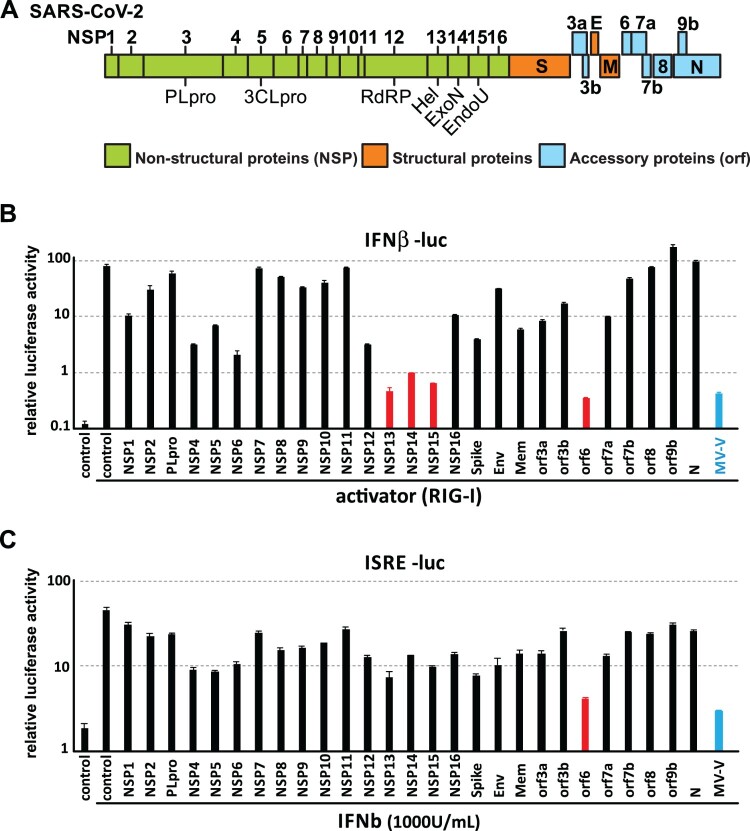
Screening of interferon antagonists among 27 SARS-CoV-2 viral proteins. (A) Genome architecture of SARS-CoV-2. (B-C) Screening of SARS-CoV-2 interferon antagonists. 293FT cells were co-transfected with (B) IFNβ-luc or (C) ISRE-luc reporter together with pRL-TK plasmid, expression vector for RIG-I 2CARD domain, and expression plasmid for one of the 27 SARS-CoV-2 viral proteins as indicated. 24-h post-transfection, cells were lysed for dual luciferase assay. IFNβ-luc: interferon beta promoter-driven firefly luciferase reporter. ISRE-luc: interferon-stimulated response element-driven firefly luciferase reporter. pRL-TK: HSV-thymidine kinase promoter-driven *Renilla* luciferase reporter. MV-V: Measles virus V protein.

Human coronavirus possesses the largest genome among all RNA viruses, encoding more than 25 viral proteins. The newly emerged SARS-CoV-2 virus encodes 27 proteins, including 16 non-structural proteins, 3 structural proteins and 8 accessory proteins (Figure 1(A)) [1]. It is well-recognized that human betacoronaviruses like SARS-CoV and MERS-CoV encode multiple interferon antagonists for effective evasion of host innate immune activation [14]. However, the key interferon antagonists of the recently emerged SARS-CoV-2 virus have yet been identified. In order to characterize the interferon antagonism of this new virus, a full panel of expression plasmids encoding 27 individual codon-optimized SARS-CoV-2 viral proteins was constructed for interferon antagonist screening. The expression of viral proteins was first confirmed by western blotting (Figure S1A). As expected, different viral proteins were expressed at various levels. Most of the viral proteins showed expression at expected size, although nsp4, nsp6 and membrane proteins appeared to form aggregates in western blotting conditions. Expression of nsp13 and nsp14 was confirmed by immunostaining (Figure 4(A)). Expression of nsp9, nsp11 and orf3b was verified by RT–PCR (data not shown).

To screen for putative SARS-CoV-2-encoded interferon antagonists, N-terminal RIG-I, a well-known upstream activator of typical interferon signalling, was used as the potent inducer for interferon production. The degree of interferon induction and suppression was assessed by quantitation of promoter activity of human interferon-beta gene (Figure 1(B)). Using this system, we found that expression of multiple SARS-CoV-2 viral proteins were able to inhibit primary interferon production to various degrees. Among all 27 SARS-CoV-2 proteins, orf6 is the most potent interferon antagonists as evidenced by more than 100-fold reduction of interferon-beta promoter activity. In addition, SARS-CoV-2 nsp13, nsp14 and nsp15 also inhibited interferon production as effective as those of orf6 and the well-known potent interferon antagonist measles virus V protein (Figure 1(B)). It is noted that SARS-CoV papain-like protease (PLpro) is one of the key interferon antagonists. Infection of a mutant SARS-CoV with the substitution of a bat PLpro in interferon-competent cells showed the loss of anti-interferon ability independent of PLpro protease activity [32]. However, SARS-CoV-2 PLpro did not show obvious inhibition on interferon production and interferon signalling in our screening (Figure 1(B,C)). In addition to inhibition of primary interferon production, the ability of the viral protein panel to inhibit interferon-stimulated gene (ISG) transcription upon treatment with recombinant interferon beta protein has also been characterized. Among all, SARS-CoV-2 orf6 exhibited the strongest inhibitory effect, to a comparable extent as that of the measles virus V protein (Figure 1(C)).

### SARS-CoV2 orf6 is a potent interferon antagonist

**Figure 2.**
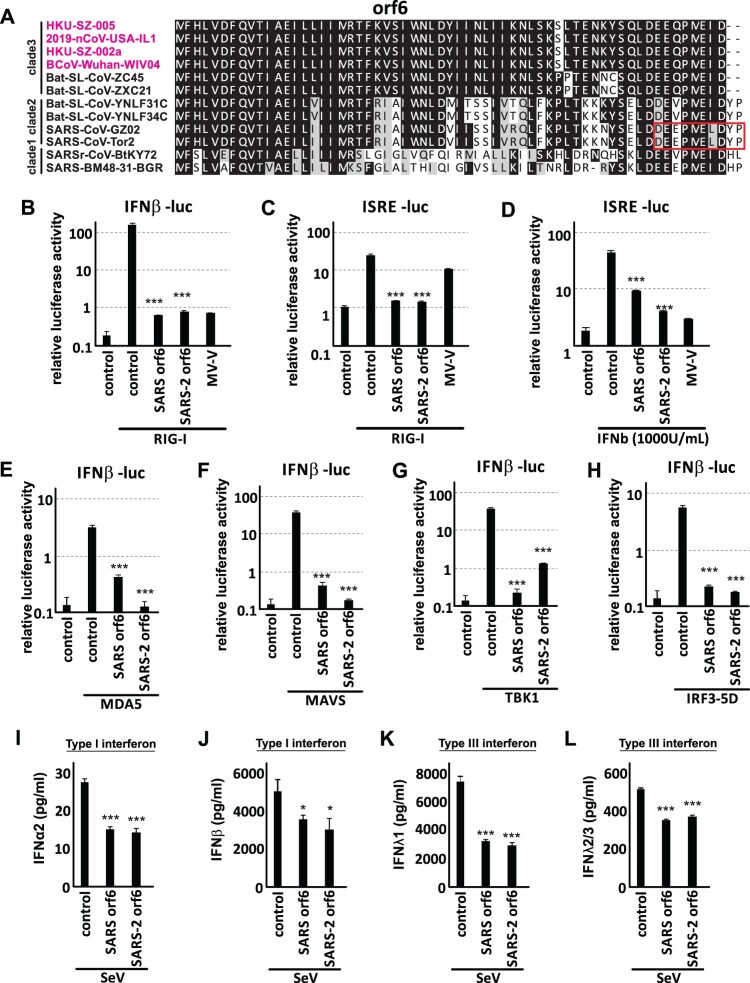
SARS-CoV-2 orf6 is one of the most potent interferon antagonists. (A) Multiple alignment of orf6. Amino acid sequences of orf6 SARS-CoV-2, SARS-CoV and SARS-like bat coronavirus were aligned using Clustal Omega. Red box indicates the region reported important for interferon antagonism. Accession numbers of the selected strains are as follow: HKU-SZ-005 (MN975262). 2019-nCoV-USA-IL1 (MN988713.1). HKU-SZ-002a (MN938384). BCoV-Wuhan-WIV04 (EPI_ISL_402124). Bat-SL-CoV-ZC45 (MG772933). Bat-SL-CoV-ZXC21 (MG772934). Bat-SL-CoV-YNLF31C (KP886808). Bat-SL-CoV-YNLF34C (KP886809). SARS-CoV-GZ02 (AY390556). SARS-CoV-Tor2 (AY274119). SARSr-CoV-BtKY72 (KY352407.1). SARS-BM48-31-BGR (GU190215). (B-C) SARS-CoV and SARS-CoV-2 orf6 inhibit RIG-I-activated interferon signalling. (B) IFNβ-luc or (C) ISRE-luc reporter was co-transfected with pRL-TK plasmid, expression vector for RIG-I 2CARD domain and expression vector for either SARS-CoV or SARS-CoV-2 orf6 into 293FT cells for 24 h. Cells were then lysed for dual luciferase assay. (D) Both SARS-CoV and SARS-CoV-2 orf6 inhibit interferon-stimulated gene transcription. ISRE-luc reporter was co-transfected with pRL-TK plasmid together with expression plasmid for orf6 of either SARS-CoV or SARS-CoV-2. 24 h post-transfection, cells were treated with 1000 units of recombinant human IFNβ for 12 h before lysis. (E-H) SARS-CoV and SARS-CoV-2 orf6 inhibit IFNβ production at a step post-IRF3 phosphorylation. IFNβ-luc, pRL-TK and expression plasmid of either SARS-CoV orf6 or SARS-CoV-2 orf6 were co-transfected into 293FT cells together with expression plasmid for either (E) MDA5, (F) MAVS, (G) TBK1, or (H) IRF3-5D, which is a phospho-mimic of activated form of IRF3. Cells were lysed at 24 h post-transfection for dual luciferase measurement. (I-L) SARS-CoV and SARS-CoV-2 orf6 inhibit secreted type I and type III interferon proteins. 293FT cells were mock transfected or transfected with SARS-CoV orf6 or SARS-CoV-2 orf6 expression plasmid. 24 h post-transfection cell were infected by 64 HA units of Cantell strain Sendai virus. Cell supernatant was collected 24 h post-infection for quantitation of (I) IFNα2, (J) IFNβ, (K) IFNλ1 and (L) IFNλ2/3 proteins using multiplex bead-based cytokine flow cytometry. Statistical significance was calculated by unpaired two-tailed student’s t-test. * represents *P *< 0.1, *** represents *P *< 0.001. Error bars denote standard deviation.

Betacoronavirus belongs to the family of Coronaviridae under the order of Nidoviridae. Within this genus, it is further divided into four sub-genera: Embecovirus (lineage A), Sarbecovirus (lineage B), Merbecovirus (lineage C) and Nobecovirus (lineage D). The newly emerged SARS-CoV-2, as well as the SARS-CoV and SARS-related (SARSr)-CoV all belong to Sarbecovirus. SARS-CoV-2 orf6 is a small protein of approximately 7 kDa. It is well-conserved within clade 3 of Sarbecovirus, but less when compared to clade 1 and clade 2 of Sarbecovirus that includes SARS-CoV and SARS-related bat coronavirus respectively (Figure 2(A)). The amino acid sequence of SARS-CoV-2 orf6 only showed 69% homology with its SARS-CoV counterpart plus two amino acids deletion at C-terminus. Previous report mapped 10 key amino acid residues (red box in Figure 2(A)) important for the interferon antagonism [24]. Although only four amino acids are conserved between SARS-CoV and SARS-CoV-2, SARS-CoV-2 orf6 remained functional in suppressing primary interferon production and interferon signalling (Figure 1(B,C)). Orf6 has been characterized as the key interferon antagonist of SARS-CoV [21,24]. The loss of orf6 rendered the virus interferon-stimulating. It is therefore worthwhile to compare the potency of interferon antagonism of orf6 from both viruses. Protein expression of orf6 of both SARS-CoV and SARS-CoV-2 was first confirmed by western blotting (Figure S1B). Interferon beta luciferase assay showed that orf6 of both viruses were able to effectively inhibit RIG-I induced interferon production (Figure 2(B)). Both of them also quelled ISG induction upon RIG-I activation or interferon-beta treatment to comparable levels (Figure 2(C,D)). In addition to activation by RIG-I, both SARS-CoV and SARS-CoV-2 orf6 prevented interferon induction activated by various signalling molecules MDA5, MAVS, TBK1 and IRF3-5D, which is a phospho-mimic of the activated form of IRF3 (Figure 2(E–H)). This suggests that orf6 of both viruses may interfere with primary interferon production at a step(s) post-IRF3 phosphorylation along the RIG-I-like receptor (RLR)-TBK1-IRF3 axis. The interferon-antagonistic function of orf6 was further supported by its inhibition of endogenous interferon beta and ISG mRNA expression upon RIG-I activation (Figure S2). Moreover, we used Cantell strain Sendai virus as an interferon inducer that mimics a real viral infection. Orf6 of both SARS-CoV and SARS-CoV-2 were able to inhibit type I (IFNα2 and IFNβ) and type III (IFNλ1 and IFNλ2/3) interferons secretion into cell culture supernatant upon Sendai virus infection (Figure 2(I–L)). These altogether supported the notion that SARS-CoV-2 is a potent interferon antagonist.

### Loss of interferon-antagonizing and deubiquitinating activity of PLpro

**Figure 3.**
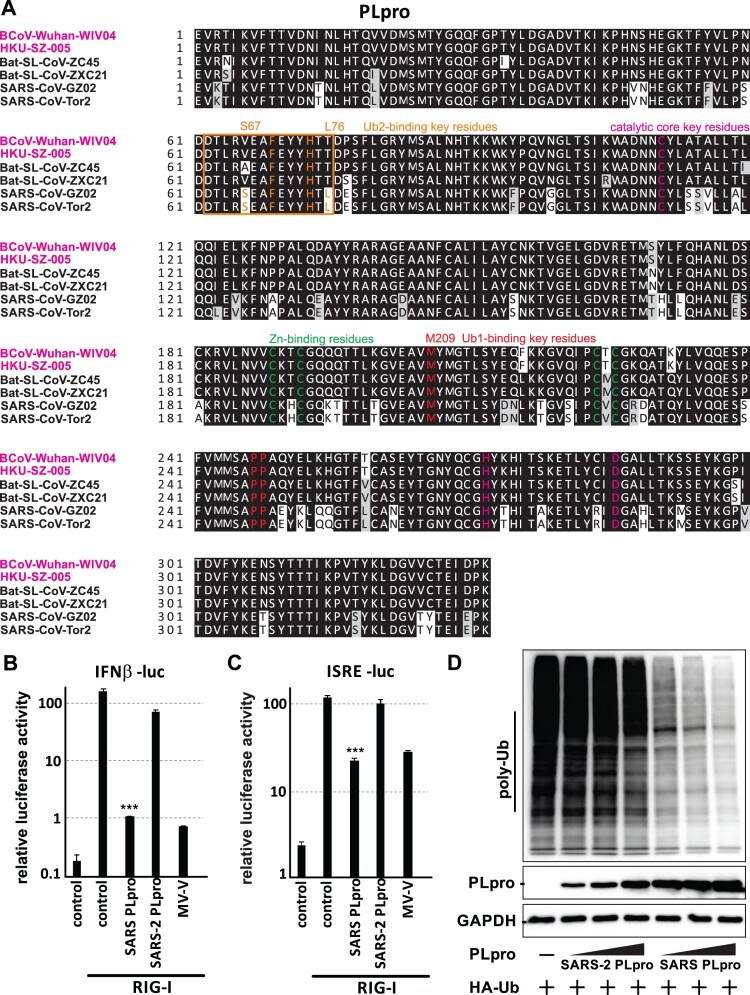
Loss of interferon antagonizing and deubiquitinase activities of SARS-CoV-2 PLpro. (A) Multiple alignment of PLpro from SARS-CoV-2, SARS-like bat coronavirus and SARS-CoV. Amino acid sequence of PLpro from selected SARS-related viruses were aligned using Clustal Omega. Pink: key residues in catalytic core. Green: residues involved in zinc binding. Red: residue for Ub1 binding. Orange: residues important for Ub2 binding. (B-C) PLpro of SARS-CoV but not SARS-CoV-2 inhibits primary interferon production. IFNβ-luc, pRL-TK, expression plasmid of RIG-I 2CARD domain and that of either SARS-CoV or SARS-CoV-2 PLpro were co-transfected into 293FT cells for 24 h for dual luciferase assay. (D) Reduced deubiquitinase activity of SARS-CoV-2 PLpro compared to SARS-CoV PLpro. HA-Ub expression plasmid was co-transfected with empty vector, or expression plasmid for FLAG-tagged SARS-CoV-2 or SARS-CoV-2 PLpro in increasing dose of 0.5, 1 and 2 µg. Cells were lysed 24 h post-transfection for western blotting using anti-HA-tag, anti-FLAG-tag and anti-GAPDH antibodies. Statistical significance was calculated by unpaired two-tailed student’s t-test. *** represents *P *< 0.001. Error bars denote standard deviation.

Betacoronavirus including Sarbecovirus (e.g. SARS-CoV and SARS-CoV-2) and Merbecovirus (e.g. MERS-CoV) encodes two functionally conserved viral proteases, the papain-like protease (PLpro) and 3C-like protease (3CLpro). The two proteases cleave viral polyproteins pp1a and pp1ab into mature forms. Other than proteolytic activity, PLpro of SARS-CoV and MERS-CoV also acts as deubiquitinase (DUB) and interferon antagonist [19,34–37]. Surprisingly, our interferon antagonist screening demonstrated that PLpro of SARS-CoV-2 marginally inhibited primary interferon production and interferon signalling (Figure 1(B,C)), despite there was protein expression of SARS-CoV-2 PLpro (Fig. S1A). Sequence analysis between SARS-CoV and SARS-CoV-2 PLpro showed that the two proteins share 83% homology in amino acid. Key amino acids important for protease function, including those in the catalytic core and zinc-binding domain, are also conserved (Figure 3(A)). To further confirm the loss of interferon-antagonizing property of SARS-CoV-2 PLpro, we compared the interferon-antagonizing function of PLpro between SARS-CoV-2 and SARS-CoV. Both proteins were confirmed to expressed by western blotting, despite SARS-CoV PLpro expressed slightly higher (Figure S1C). As expected, SARS-CoV PLpro was able to inhibit both RIG-I-induced interferon production and interferon signalling as potent as measles V protein. However, minimal inhibitory effect of SARS-CoV-2 PLpro could be observed (Figure. 3(B,C)). The loss of interferon antagonism was further evidenced by the inability of SARS-CoV-2 PLpro to inhibit induction of endogenous interferon beta and ISG mRNA expression upon RIG-I activation (Figure S2). Next, we compared the DUB activity between SARS-CoV PLpro and SARS-CoV-2 PLpro. It is believed that viral deubiquitinating enzymes might be one of the key determinants of viral pathogenesis. SARS-CoV PLpro possesses two ubiquitin-binding domains, Ub1 and Ub2, for the binding of K48-linked and K63-linked ubiquitin chains [38]. From the multiple sequence alignment analysis, we found that the key residues of Ub1 domain (e.g. M209) is conserved among SARS-CoV-2, SARS-CoV and SARS-related CoV. However, the key residues of Ub2 domain (S67 and L76) of SARS-CoV are different from that of SARS-CoV-2. We therefore speculated that the DUB function of SARS-CoV-2 PLpro might also be altered. As expected, we showed that expression of SARS-CoV PLpro, but not SARS-CoV-2 PLpro, strongly deubiquitinated host polyubiquitinated proteins (Figure 3(D)). Such discrepancy is unlikely due to difference in protein expression level of the two PLpro, since a drastic difference in protein polyubiquitination could still be observed even when the plasmid transfection dose was adjusted to yield similar PLpro expression level (Figure 3(D), lane 4 and 5).

### SARS-CoV-2 nsp13, nsp14, nsp15 and orf6 inhibit nuclear localization of IRF3

**Figure 4.**
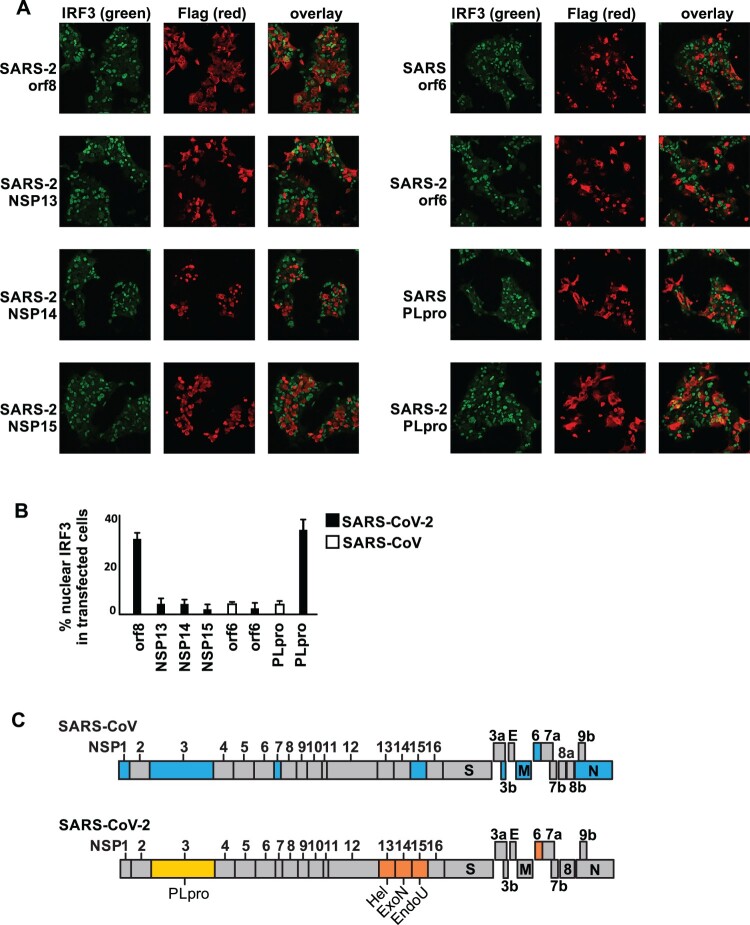
SARS-CoV-2 nsp13, nsp14, nsp15 and orf6, but not PLpro are interferon antagonists. (A-B) Suppression of IRF3 nuclear translocation by selected SARS-CoV-2 and SARS-CoV proteins. 293FT were transfected with the indicated overexpression plasmids. 48 h post-transfection, cells were infected with 400 haemagglutinating (HA) units of Sendai virus (Cantell strain) for 6 h, followed by 4% paraformaldehyde fixation and immunostaining with anti-FLAG and anti-IRF3 antibodies. Images were acquired using confocal microscope (A). Green: IRF3; Red: viral proteins. Percentage of nuclear IRF3-positive transfected cells was counted from three fields of view (B). (C) Schematic diagram showing SARS-CoV and SARS-CoV-2 interferon antagonists. The genome architecture of SARS-CoV (upper panel) and SARS-CoV-2 (lower panel) are depicted. The SARS-CoV interferon antagonists previously reported are highlighted blue. SARS-CoV-2 interferon antagonists identified in this study are highlighted orange, with PLpro that has compromised interferon antagonising and DUB activity highlighted yellow. Hel: helicase; ExoN: exonuclease; EndoU: endoribonuclease; PLpro: papain-like protease.

In the co-expression screening, nsp13, nsp14, nsp15 and orf6 showed the strongest inhibitory effect on interferon production upon RIG-I activation (Figure 1). The inhibition was also confirmed by quantitation of endogenous interferon beta and ISG transcript expression (Figure S2). Here we further examined their ability to inhibit nuclear localization of IRF3, which is the key transcription factor driving interferon beta transcription, upon Sendai virus infection. Cantell strain of Sendai virus is commonly used as a potent interferon inducer. It contains more than 95% defective interfering virus and its viral defective genome has been shown to activate both TLR- and RLR-mediated interferon signalling. Here, we used Sendai virus as a model that mimics real viral infection and examined the anti-interferon activity of these four viral interferon antagonists (Figure 4(A,B)). While the negative control SARS-CoV-2 orf8, which showed no interferon antagonizing effect in our screening, could not inhibit IRF3 nuclear localization; nsp13, nsp14, nsp15 could all quell IRF3 nuclear localization. Similarly, orf6 of both SARS-CoV-2 and SARS-CoV could inhibit IRF3 nuclear localization. However, PLpro of SARS-CoV but not SARS-CoV-2 had the inhibitory effect. Altogether, we demonstrated that SARS-CoV-2 helicase nsp13, exonuclease nsp14, endoribonuclease nsp15 and accessory protein orf6 are the strongest interferon antagonists, while PLpro, unlike its SARS-CoV counterpart, lost both DUB and interferon-antagonizing activity (Figure 4(C)).

## Discussion

In this study we search for potential viral interferon antagonists of SARS-CoV-2. Similar to other highly pathogenic human coronaviruses, such as SARS-CoV and MERS-CoV, of which a number of viral interferon antagonists have been reported, we found that several SARS-CoV-2 viral proteins possess the ability to inhibit primary interferon production (Figure 1). However, it is intriguing to unveil the reasons for coronaviruses to encode more than one interferon antagonist and whether all of them can function as potent interferon antagonists during infection. While this study provided valuable information about individual SARS-CoV-2 protein’s potential to inhibit host interferon response, a few points have to be noted. First, the interferon-antagonizing properties of a viral protein may differ between real infection and in in-vitro settings. Second, expression level of the individual viral protein during infection can differ a lot from overexpression, which in turn affects the magnitude of interferon suppression. Third, altered localization of viral proteins during infection may affect their activities. For instance, nsp13, 14 and 15 are recruited to double membrane vesicles (DMVs) during viral infection. The sequestration of these viral interferon antagonists to specialized compartments may prevent them from efficiently interacting with host interferon signalling molecules. This is in concordance with previous studies on SARS-CoV that although orf3b, orf6 and nucleoprotein of SARS-CoV can individually function as interferon antagonist, infection with orf6-null SARS-CoV triggered STAT1 nuclear translocation [21,24]. This implied that orf6 may be the key interferon antagonist during SARS-CoV infection, the loss of which is enough to render the virus interferon-stimulatory.

Orf6 protein is conserved within clade 2 of Sarbecovirus (lineage B of Betacoronavirus), which includes SARS-CoV (SARS-CoV GZ02 and SARS-CoV Tor2) and bat SARS-like CoV (bat-SL-CoV YNLF31C and bat-SL-CoV YNLF34C), but less when compared to clade 3 of Sarbecovirus (Figure 2(A)). SARS-CoV orf6 has been shown to inhibit primary interferon production [21] and antagonize STAT1 function (interferon signalling) by altering the nuclear import factors [24]. Although there is only 69% amino acid identity between SARS-CoV orf6 and SARS-CoV-2 orf6, we found that the two orf6 can potently suppress both primary interferon production and interferon signalling (Figures 2(B–H) and 4(A)). Since infection of SARS-CoV-2 in ex-vivo human lung tissues demonstrated stronger suppression of type-I/III interferon production when compared to SARS-CoV [8], further mechanistic studies of SARS-CoV-2 orf6 on the inhibition of primary interferon production and in-vivo investigation of orf6-deleted virus will provide important insights into the pathogenesis of SARS-CoV-2.

Nsp3 PLpro is a multifunctional viral protein. One of the main functions is as protease for cleavage of polyprotein pp1a and pp1ab of all coronaviruses [39]. It also possesses protease-independent DUB activity and interferon-antagonizing activity. The DUB activity has also been suggested to be required for the interferon-antagonizing function. Although we observed reduction in interferon-antagonizing and DUB activities of SARS-CoV-2 PLpro when compared to its SARS-CoV counterpart, the key residues in catalytic core and those for zinc-binding and Ub1-binding are conserved between the two proteins. The exact amino acid substitution(s) that causes the change in PLpro’s activity by far is unclear, but the difference in Ub2-binding residues (S67 and L76 of SARS-CoV PLpro) might partially account for this discrepancy. SARS-CoV-2 encodes multiple putative interferon antagonists. The loss of interferon-antagonizing activity of PLpro may not result in drastic difference in interferon antagonism of SARS-CoV-2. However, how the loss of DUB activity, which is functionally unique to PLpro among all 27 SARS-CoV-2 viral proteins, would affect infection and the subsequent pathology warrants further study.

## Supplementary Material

Supplemental Material
