# Supplementary material for: SARS-CoV-2 nsp13, nsp14, nsp15 and orf6 function as potent interferon antagonists
Source: Emerg Microbes Infect. 2020 Jun 20;9(1):1418–28. doi: 10.1080/22221751.2020.1780953 (PMC7473193; doi:10.1080/22221751.2020.1780953)
Supplement: Supplemental Material [file TEMI_A_1780953_SM1758.zip › Supplementary files/Figure captions.docx]

**Figure S1. Western blotting of SARS-CoV-2 and SARS-CoV viral proteins**

**(A)** Overexpression of individual SARS-CoV-2 protein. 293FT cells were transfected with overexpression plasmids for the indicated flag-tagged viral proteins. Cells were lysed for western blotting 48hr post-transfection using anti-FLAG-tag and anti-beta-actin antibodies. **(B-C)** Expression of PLpro and orf6 of SARS-CoV-2 and SARS-CoV. EV: empty vector.

**Figure S2. SARS-CoV-2 nsp13, nsp14, nsp15 and orf6 inhibited IFNβ and ISG mRNA expression**

Repression of **(A)** IFNβ, **(B)** ISG15 and **(C)** IFITM3 transcript expression by SARS-CoV-2 nsp13, nsp14, nsp15 and orf6. Expression plasmid for RIG-I 2CARD domain was co-transfected with indicated SARS-CoV-2 or SARS-CoV expression plasmids into 293FT cells. Cells were collected at 24hr post-transfection for RNA extraction and endogenous transcript levels of the indicated genes were quantified by qPCR. Statistical significance was calculated by unpaired two-tailed student’s t-test. *** represents P<0.001. Error bars denote standard deviation.
